# Efficient gene transfer into T lymphocytes by fiber-modified human adenovirus 5

**DOI:** 10.1186/s12896-019-0514-x

**Published:** 2019-04-24

**Authors:** Yun Lv, Feng-Jun Xiao, Yi Wang, Xiao-Hui Zou, Hua Wang, Hai-Yan Wang, Li-Sheng Wang, Zhuo-Zhuang Lu

**Affiliations:** 1 Graduate School of Anhui Medical University, 81 Meishan Road, Shu Shan Qu, Hefei, Anhui People’s Republic of China; 2 Department of Experimental Hematology, Beijing Institute of Radiation Medicine, 27 Taiping Road, Beijing, China; 3 State Key Laboratory of Infectious Disease Prevention and Control, National Institute for Viral Disease Control and Prevention, Chinese Center for Disease Control and Prevention, 100 Ying Xin Jie, Beijing, China; 4 Affiliated Hospital of Qingdao University, 16 JiangSu Road, Qingdao, People’s Republic of China

**Keywords:** Gene therapy, Adenovirus 5 vector, Human hematopoietic cells

## Abstract

**Background:**

The gene transduction efficiency of adenovirus to hematopoietic cells, especially T lymphocytes, is needed to be improved. The purpose of this study is to improve the transduction efficiency of T lymphocytes by using fiber-modified human adenovirus 5 (HAdV-5) vectors.

**Results:**

Four fiber-modified human adenovirus 5 (HAdV-5) vectors were investigated to transduce hematopoietic cells. F35-EG or F11p-EG were HAdV-35 or HAdV-11p fiber pseudotyped HAdV-5, and HR-EG or CR-EG vectors were generated by incorporating RGD motif to the HI loop or to the C-terminus of F11p-EG fiber. All vectors could transduce more than 90% of K562 or Jurkat cells at an multiplicity of infection (MOI) of 500 viral particle per cell (vp/cell). All vectors except HR-EG could transduce nearly 90% cord blood CD34+ cells or 80% primary human T cells at the MOI of 1000, and F11p-EG showed slight superiority to F35-EG and CR-EG. Adenoviral vectors transduced CD4+ T cells a little more efficiently than they did to CD8+ T cells. These vectors showed no cytotoxicity at an MOI as high as 1000 vp/cell because the infected and uninfected T cells retained the same CD4/CD8 ratio and cell growth rate.

**Conclusions:**

HAdV-11p fiber pseudotyped HAdV-5 could effectively transduce human T cells when human EF1a promoter was used to control the expression of transgene, suggesting its possible application in T cell immunocellular therapy.

## Background

T lymphocytes play an important role in adaptive immunity. It is highly desirable to introduce exogenous wild type or mutant genes into primary T cells for studying their growth, differentiation, death, and interaction with other immunocytes [1]. T cells are also important targets for gene therapy of numerous human diseases, including cancer, diabetes, arthritis and AIDS [2–5]. Gene delivery to T cells has been achieved by retroviruses including gamma-retrovirus, lentivirus and alpha-retrovirus [2, 6]. Retrovirus is able to integrate into the host’s genome, and the transgene stably expresses. However, retrovirus based vector has drawbacks: it can hardly be produced on a large scale; purification operation cannot substantially improve its performance and is often skipped; transfection efficiency is relatively low; and the integration property can possibly cause genetic toxicity such as transformation of host cells.

Adenoviral vector is widely used in gene therapy and vaccine development [7–9]. Adenovirus is non-integrating vector. It can be amplified to very high titer and be conveniently purified. The cloning capacity is higher and the expression of exogenous gene is relatively more efficient. When combined with other gene transfer technique such as transposon, EBV nuclear antigen 1 (EBNA-1) or scaffold/matrix attachment region (S/MAR) element, adenovectors can be used to stably express transgene [10–12]. There are needs to transiently transduce T cells, such as genome editing of disease associated gene [13, 14]. However, adenovirus is seldom used in transducing T cells due to the low gene transfer efficiency.

Adenoviruses, containing a genome of double-stranded DNA, are nonenveloped icosahedral particles with fibers projecting from the vertices. Human adenoviruses (HAdV) belong to the genus of mastadenovirus and are divided into A-G species [15, 16]. The commonly-used adenovectors are constructed based on HAdV-5, a type of HAdV-C. HAdV-5 attaches host cell through the interaction of viral fiber with primary cellular receptor CAR. Binding of the RGD motif of penton base to coreceptor integrin (avb3 or avb5) further facilitates virus entry to cells by triggering endocytosis [17].

Hematopoietic cells including T cells can be poorly transduced by HAdV-5 vectors due to paucity of CAR on the cellular surface [17–19]. HAdV-35, a type of HAdV-B, uses CD46 as the cellular receptor, which is extensively expressed in hematopoietic cells. Therefore, HAdV-35 is able to infect human hematopoietic cells. Adenoviral fiber consists of three domains of knob, shaft and tail. Knob recognizes and binds to the cellular receptor, tail interacts with the penton base and makes the fiber implant in virion, and the shaft links knob and fiber. Adenoviral vector system of HAdV-5 has been extensively studied and is the most robust system. In order to construct adenovirus vector that can infect hematopoietic cells, the fiber knob and shaft domains of HAdV-5 was replaced with that of HAdV-35 and the resulted adenovirus was called HAdV-5F35, which could be rescued and amplified conveniently with the HAdV-5 vector system [20, 21]. HAdV-11p, another type of HAdV-B, is different from HAdV-35 in that HAdV-11p binds to both CD46 and DSG-2 on the surface of host cells [22, 23]. It was reported that HAdV-11p was more efficient than HAdV-35 when infecting CD34+ hematopoietic cells [24]. We constructed HAdV-5F11p vectors [25]. However, HAdV-5F35 and HAdV-5F11p have not be systematically compared in the ability of gene delivery to human hematopoietic cells, especially T cells. Insertion of RGD motif to the HI loop of HAdV-5 fiber knob domain could improve the ability of HAdV-5 to infect T cells [26–28]. In this study, we attempted to evaluate the gene transfer efficiency of HAdV-5F35 and HAdV-5F11p and to access whether insertion of RGD motif could further improve the capability of HAdV-5F11p.

## Results

### Construction of adenoviral vectors

**Fig. 1:**
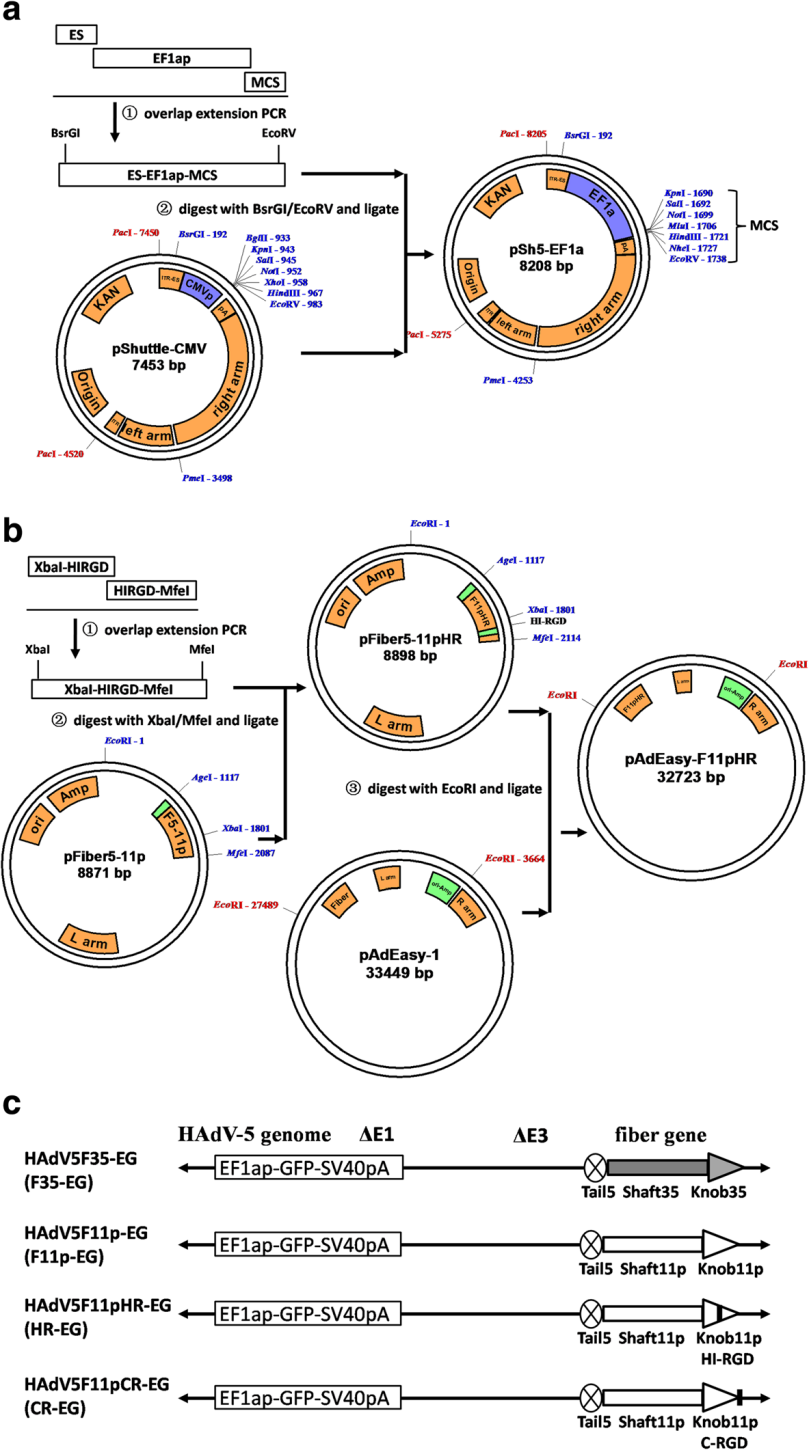
Schematic diagram of the construction of fiber-modified human adenovirus 5 (HAdV-5) vectors. **a** Construction of the shuttle plasmid carrying human EF1a promoter. **b** Construction of the backbone plasmid carrying modified fiber gene. **c** Fiber-modified HAdV-5 vectors.All vectors contained the same human EF1a promoter controlled GFP expression cassette inserted into the E1 region and differently modified fiber genes. CMVp, CMV promoter; C-RGD, RGD4C fused to the C-terminus of HAdV-11p fiber; EF1ap, human EF1a promoter; ES, encapsidation signal; HI-RGD, RGD4C inserted into the HI loop of HAdV-11p fiber Knob; ITR, inverted terminal repeat; Knob11p, knob of HAdV-11p fiber; Knob35, knob of HAdV-35 fiber; MCS, multiple cloning site; pA, SV40 polyA signal; Shaft11p, shaft of HAdV-11p fiber; Shaft35, shaft of HAdV-35 fiber; Tail5, tail of HAdV-5 fiber

We constructed 5 first-generation adenovectors, in which the E1/E3 regions were deleted and GFP expression cassette including the human EF1a promoter, GFP coding sequence and SV40 polyA signal were inserted into the E1 region. The five vectors were the same except the fiber protein: HAdV5-EG contained the original fiber of HAdV-5 and served as a control vector; HAdV5F35-EG contained a chimeric fiber of HAdV-5 tail and HAdV-35 shaft and knob; HAdV5F11p-EG contained a chimeric fiber of HAdV-5 tail and HAdV-11p shaft and knob; HAdV5F11pHR-EG was different from HAdV5F11p-EG in that RGD4C peptide was inserted into the HI loop of the knob domain; and HAdV5F11pCR-EG was different from HAdV5F11p-EG in that RGD4C peptide was fused to the C-terminal of the fiber with a [GGGGS]3 linker (Fig. 1). The five vectors were abbreviated as Ad5-EG, F35-EG, F11p-EG, HR-EG and CR-EG, respectively. The genomic region that encoded the modified fiber was confirmed by DNA sequencing. All five vectors were rescued, amplified, purified and titrated. The particle-to-infectious unit ratio was 11 for Ad5-EG, 20 for F35-EG and F11p-EG, 10 for HR-EG, and 400 for CR-EG, implying various gene delivery ability on 293 cells.

### Transduction of hematopoietic cell lines

**Fig. 2:**
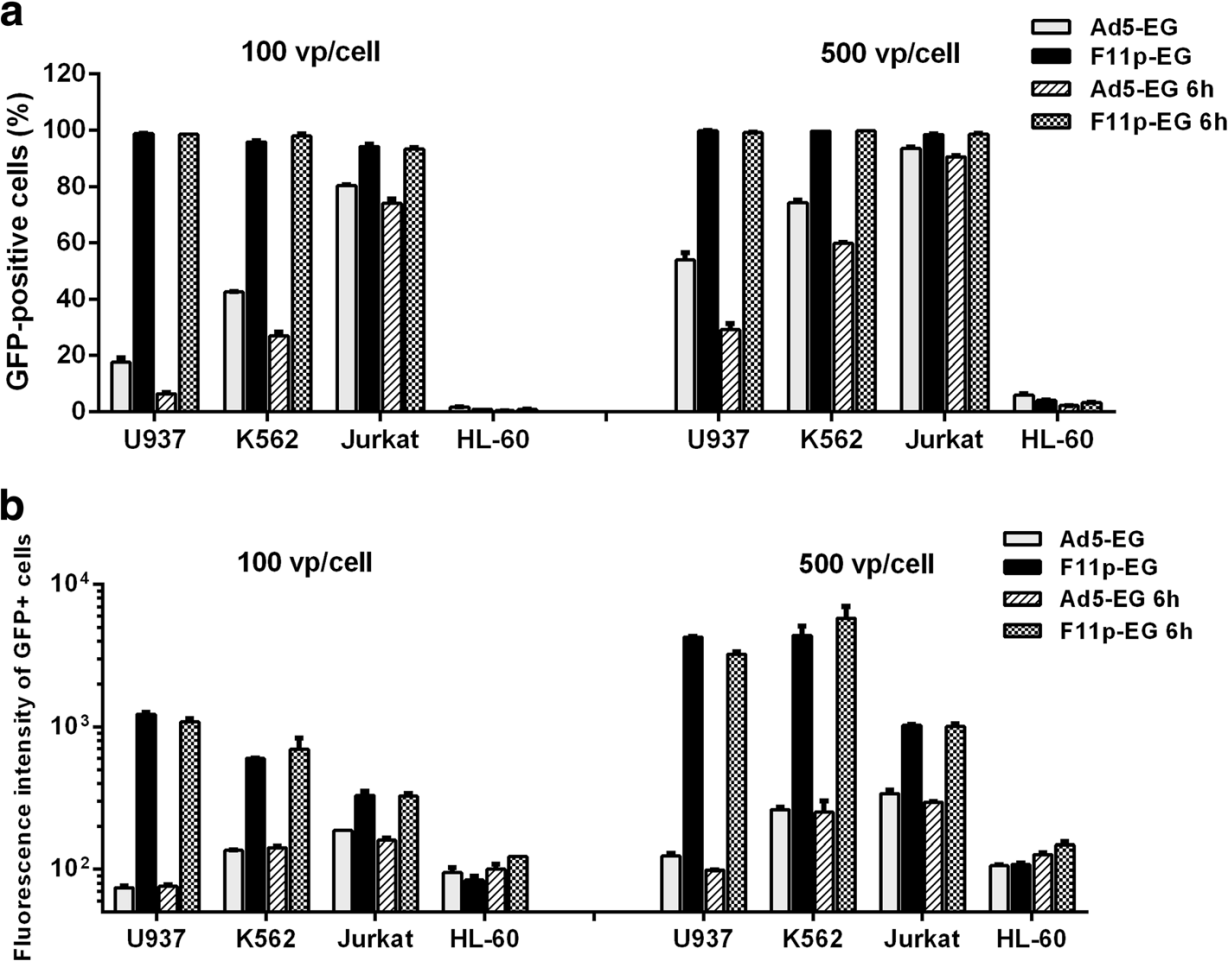
Transduction of hematopoietic cell lines by Ad5-EG and F11p-EG. Cells were infected with adenoviral vectors at MOIs of 100 or 500 vp/cell without virus removal (Ad5-EG and F11p-EG), or viruses were discarded by centrifugation and washing after 6 h’ incubation (Ad5-EG 6 h and F11p-EG 6 h). GFP expression was analyzed with flow cytometry assay 2 days post infecton. The percentage of GFP+ cells (**a**) and the mean fluorescence intensity of GFP+ cells (**b**) were compared among different vectors and cell lines

Four cell lines of U937, K562, Jurkat and HL-60 were chosen for evaluating the gene transfer ability of these adenoviral vectors. Cells were infected and the GFP fluorescence was analyzed 2 days later. The abilities of Ad5-EG and F11p-EG to transduce hematopoietic cell lines were compared firstly (Fig. 2). Both vectors could hardly transduce HL-60 cells. For U937 and K562 cells, F11p-EG was strikingly superior to Ad5-EG. For Jurkat cells, the percentages of GFP-positive cells were very close between Ad5-EG and F11p-EG groups. However, if we looked more closely, we would see the difference. When Jurkat cells were infected with F11p-EG at an MOI of 100 vp/cell, more than 94% cells were GFP-positive; and the percentage of GFP+ cells was close to 94% while Ad5-EG was used at an MOI of 500 vp/cell. When the data of the geometric mean of fluorescence intensity of GFP-positive cells were checked, the result was the same, suggesting that F11p-EG was five times more efficient than Ad5-EG on Jurkat cells. The cells were infected without virus removal, or viruses were removed after 6 h’ incubation (Fig. 2). For F11p-EG, prolonging incubation time had little influence on the transduction. In agreement with previous publications, HAdV-5 with original fiber was a poor gene transfer vector for hematopoietic cells. In order to simplify the procedure, only four vectors of F35-EG, F11p-EG, HR-EG and CR-EG were compared to find a better one for T cells transduction in following experiments.

**Fig. 3:**
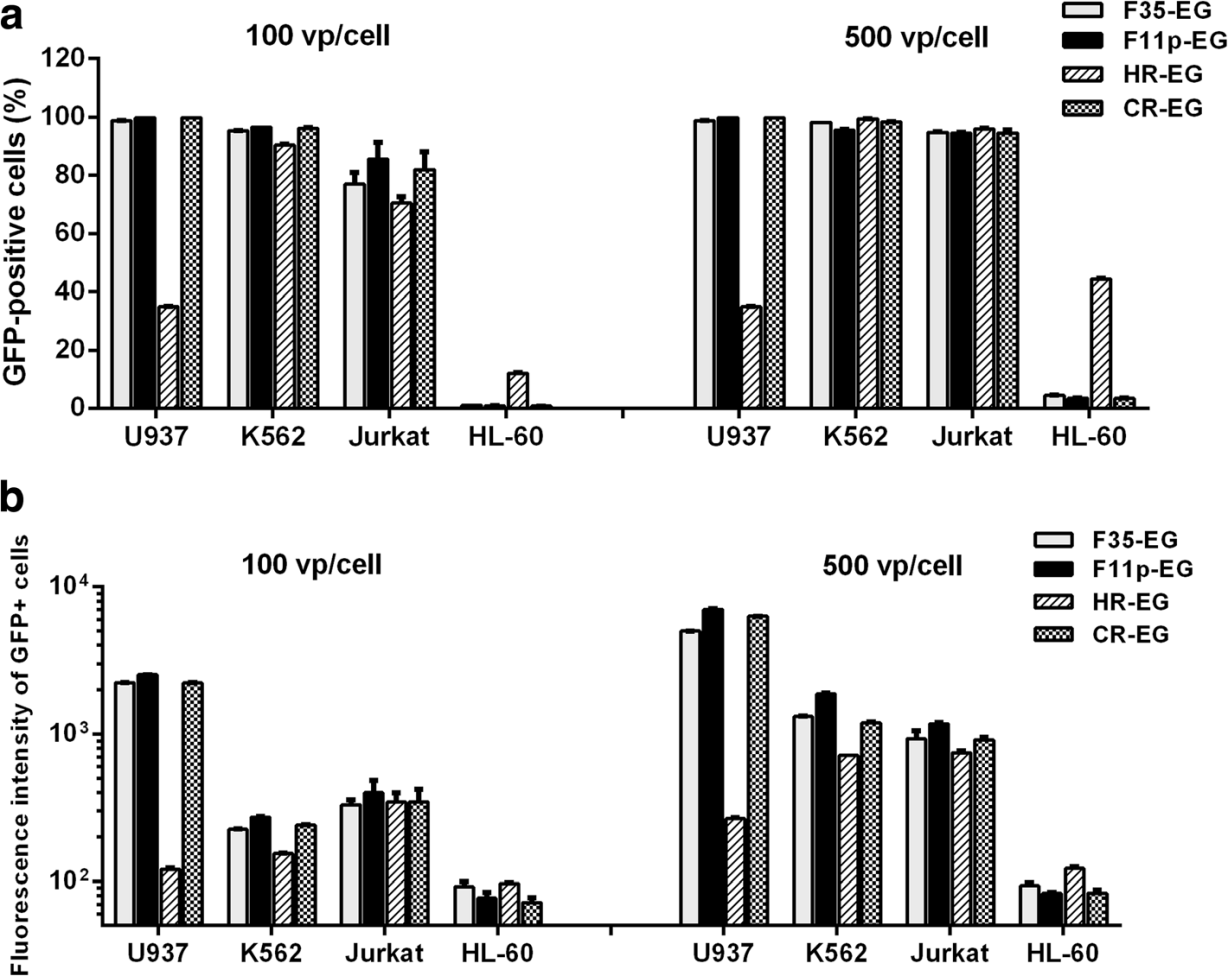
Transduction of hematopoietic cell lines by fiber-modified HAdV-5 vectors. Cells were infected with adenoviral vectors at MOIs of 100 or 500 vp/cell, and GFP expression was analyzed with flow cytometry 2 days post infecton. The percentage of GFP+ cells (**a**) and the mean fluorescence intensity of GFP+ cells (**b**) were compared among different vectors and cell lines

For the four fiber-modified HAdV-5, HL-60 is the most insensitive cell line. When the MOI was increased to 500 vp/cell, only 44% of HL-60 cells could be infected by HR-EG. For the other 3 viruses, the infection efficiency was less than 5%. Although HR-EG was the most effective vector for HL-60, it was the weakest one for U937. When infected by HR-EG at an MOI of 100 vp/cell, 35% of U937 cells was GFP-positive. However, more than 98% cells were GFP-positive when U937 was transduced by the other 3 viruses. The gene transfer ability was comparable for all 4 vectors when infecting K562 or Jurkat cells (more than 90% GFP+ cells at the MOI of 500 vp/cell), while F11p-EG had slight superiority at the MOI of 100 (Fig. 3a). To further evaluate the difference among cell lines, mean fluorescence intensity of GFP+ cells was compared. When the percentage of GFP+ cells was close to 100%, GFP had the strongest expression (highest fluorescence intensity) in U937 cells (Fig. 3b).

### Transduction of human CD34+ cord blood cells

**Fig. 4:**
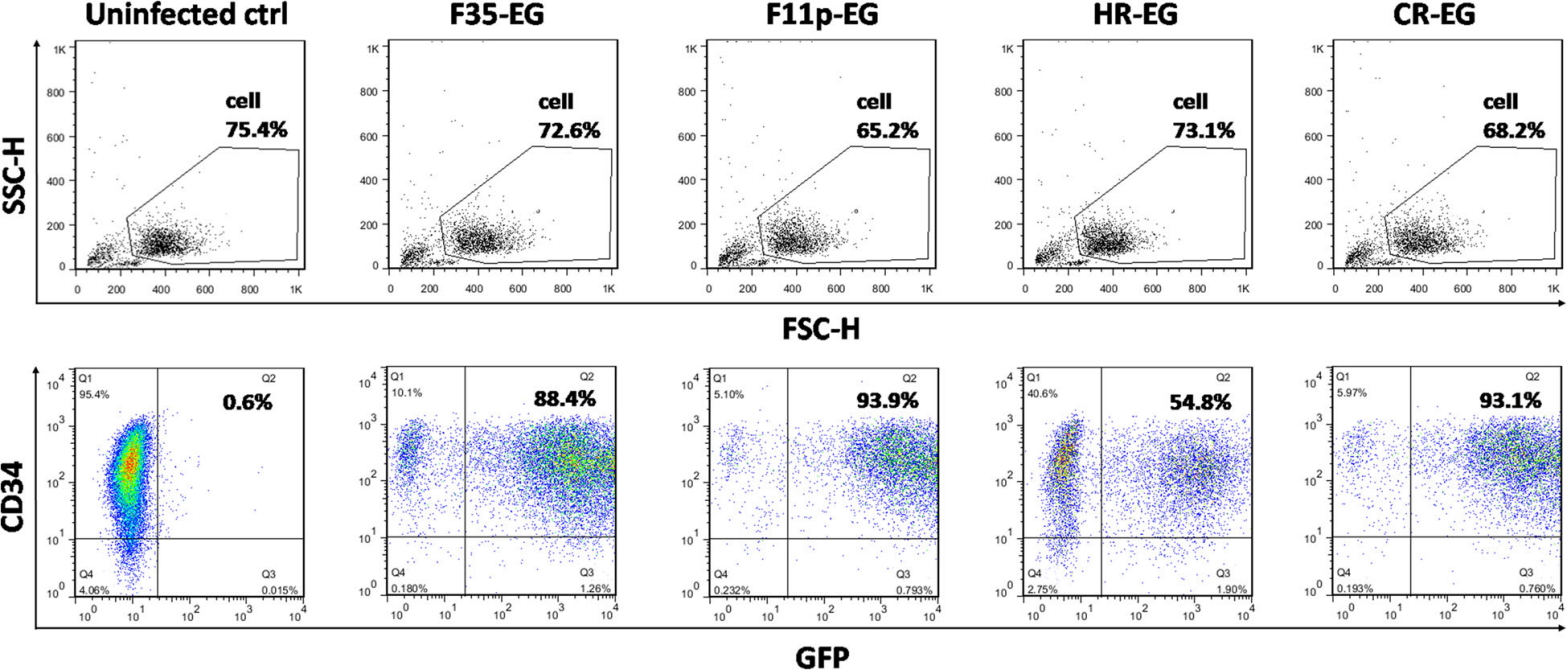
Transduction of cord blood CD34+ cells by fiber-modified HAdV-5 vectors. Isolated cord blood CD34+ cells were infected by adenoviral vectors at an MOI of 1000 vp/cell. Two days post infection, cells were labelled with APC-conjugated anti-CD34 antibody, and the GFP and APC fluorescences were analyzed with flow cytometry

CD34+ cells were isolated from cord blood mononuclear cells and infected with the 4 vectors at an MOI of 1000 vp/cell. As shown in Fig. 4, the purity of the cells was high (CD34+ cells were more than 95%), and the gene transfer efficiency was acceptable. HR-EG had the lowest efficiency of 55%, F35-EG had a gene transfer efficiency of 88%, while F11p-EG and CR-EG could transduce as much as 93% of CD34+ cells.

### Transduction of primary human T cells

**Fig. 5:**
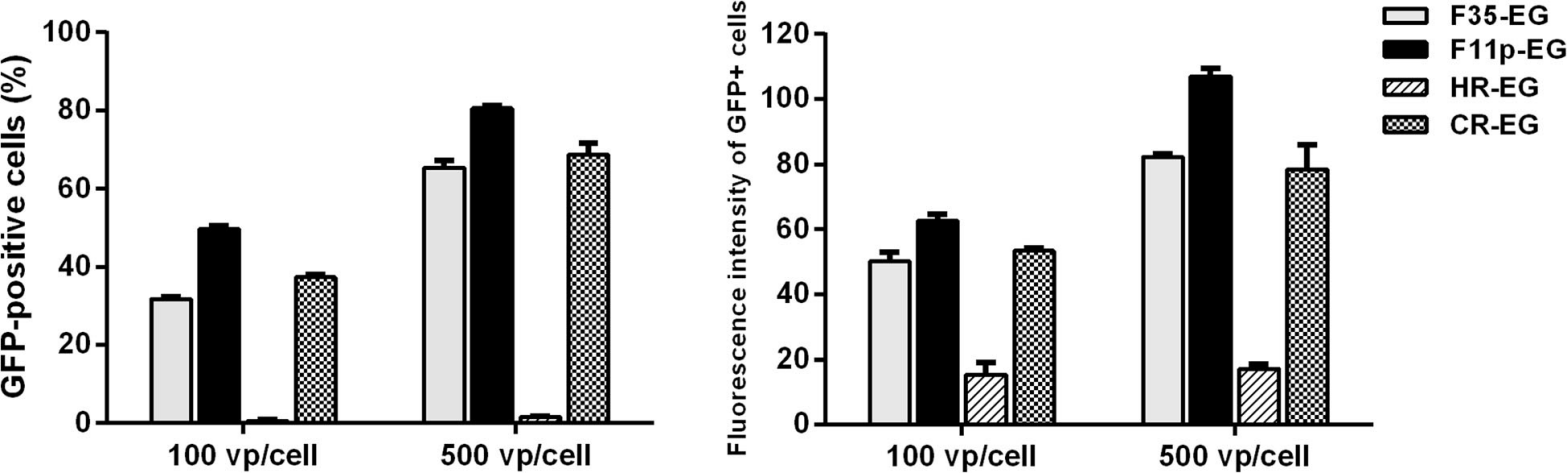
Transduction of primary human T lymphocytes by fiber-modified HAdV-5 vectors. T cells were isolated from the peripheral blood of heath donors, expanded through activation with anti-CD3 and anti-CD28 antibodies coated beads, infected by adenoviral vectors at MOIs of 100 or 500 vp/cell. GFP expression was analyzed with flow cytometry 2 days post infection. The results of the percentage of GFP+ cells and the mean fluorescence intensity of GFP+ cells were shown

HR-EG could hardly transduce primary T cells considering that less than 2% cells were GFP-positive when infected with an MOI of 500 vp/cell. F11p-EG was superior to F35-EG or CR-EG. F11p-EG could tranduce 80% T cells while F35-EG or CR-EG could infect less than 70% when an MOI of 500 vp/cell was used (Fig. 5a). Furthermore, F11p-EG-infected cells had the strongest GFP expression (Fig. 5b).

### Transduction of T cell subgroups

**Fig. 6:**
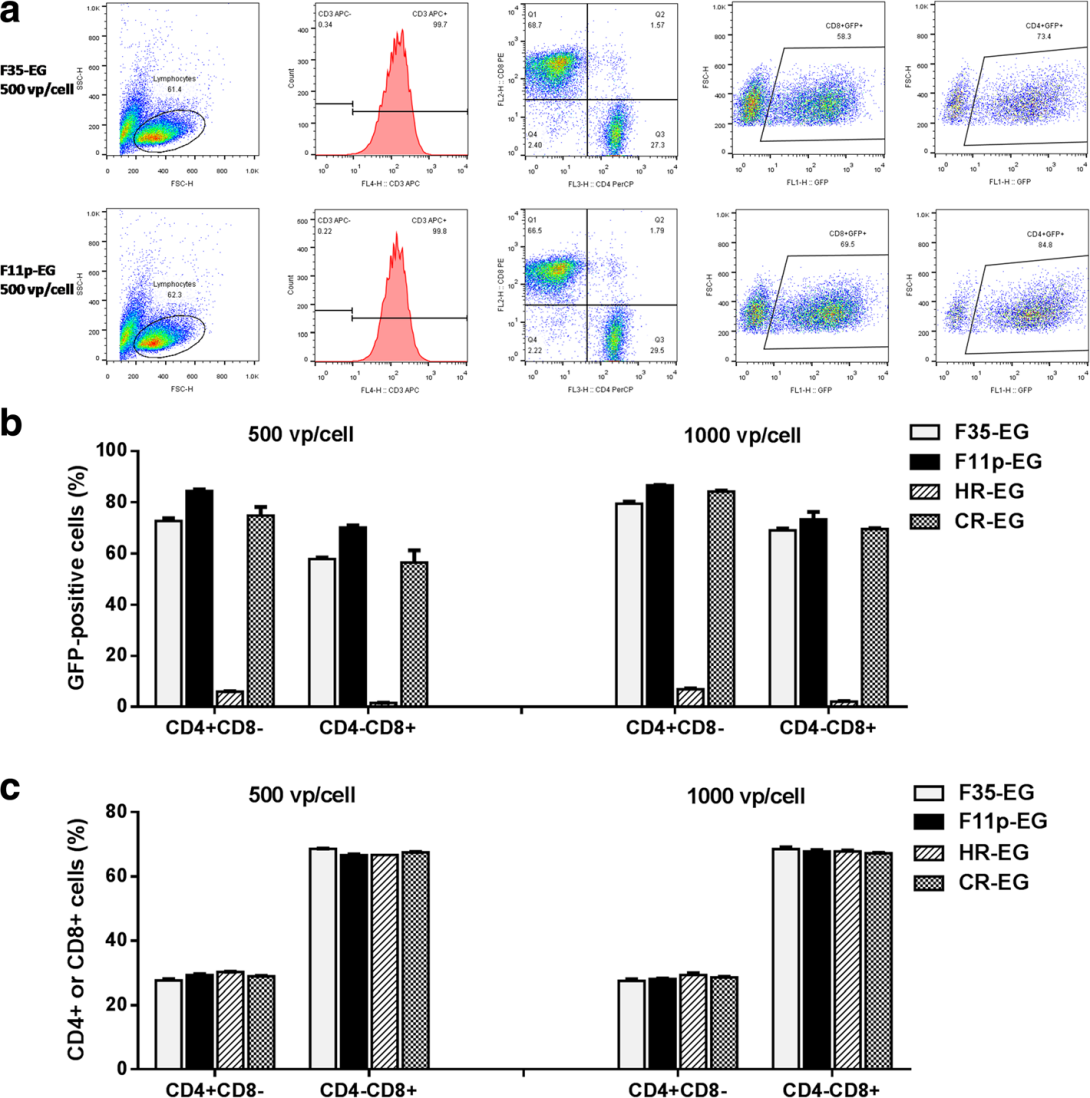
Transduction of CD4+ or CD8+ T cells by fiber-modified HAdV-5 vectors. T cells were isolated, expanded and infected by adenoviral vectors at MOIs of 500 or 1000 vp/cell. Two days post infection, cells were labelled with fluorescein-conjugated anti-CD3, anti-CD4 and anti-CD8 antibodies. GFP and fluoresceins were analyzed with flow cytometry. The data processing procedure was shown (**a**). Live lyphocytes were gated and separated from the cellular debris on the FSC-H vs. SSC-H dot plot, and CD3+ T cells were then gated and grouped according to the expression of CD4 or CD8 molecules. GFP expression in CD4 + CD8- or CD4-CD8+ subgroups were separately analyzed (**b**), and the percentages of CD4 + CD8- or CD4-CD8+ cells in CD3+ T cells were calculated (**c**)

T cells were infected by 4 vectors, respectively. Two days post infection, cells were labelled with fluorescein-conjugated anti-CD3, anti-CD4 and anti-CD8 antibodies, and analyzed with flow cytometry. As shown in Fig. 6a, lymphocytes were gated from the cellular debris on the FSC-H vs. SSC-H dot plot and analyzed for the expression of CD3. Nearly all cells were CD3-positive, indicating a high purity of cultured T cells. Gated CD3+ cells were further divided into CD4 + CD8- and CD4-CD8+ subsets, and GFP expression was separately analyzed. As shown in Fig. 6b, HR-EG could hardly transduce either CD4+ or CD8+ cells. The other 3 viruses could transduce considerable amount of T cells while F11p-EG was superior to F35-EG or CR-EG (Fig. 6b). When comparing the gene transfer efficiency between T cell subgroups, it was true for all vectors that they could transduce more CD4+ cells than CD8+ cells.

Viral infection might influence the host cell growth. Although the 4 vectors transduced the T cells with different efficiency, the percentages of CD4+ and CD8+ cells did not significantly vary among different virus-infected groups (Fig. 6c), suggestion that virus infection did not alter the inherent growth pattern of T cell subgroups. This topic was further investigated in the next paragraph.

### Effect of viral infection on the ratio of CD4/CD8 T cells and dynamic expression of transgene

**Fig. 7:**
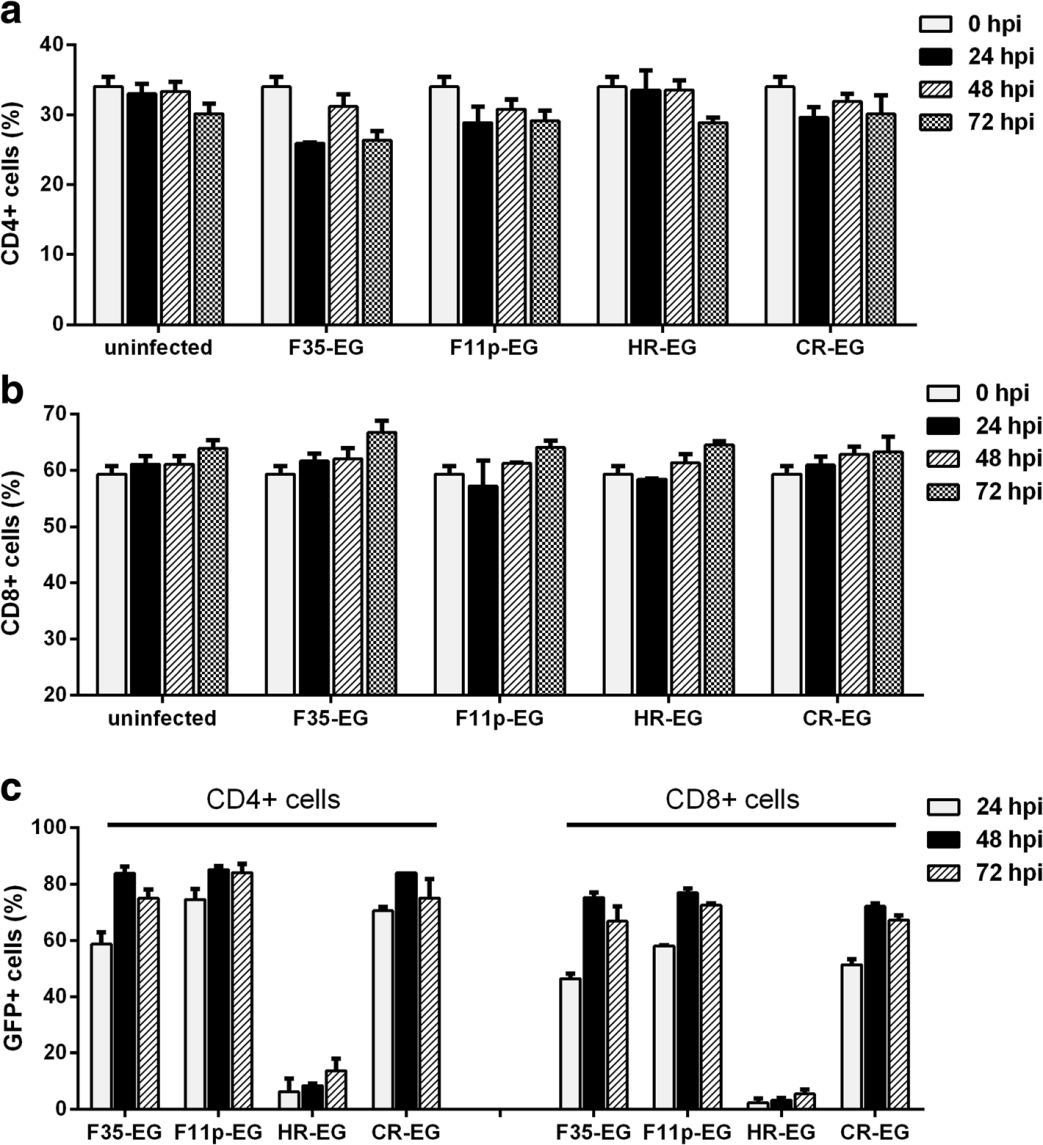
Effect of viral infection on the ratio of CD4/CD8 T cells. T cells were isolated, expanded and infected by adenoviral vectors at an MOI of 1000 vp/cell. At 0, 24, 48 and 72 h post infection (hpi), cells were harvested and labelled with fluorescein-conjugated anti-CD4 and anti-CD8 antibodies. GFP and fluoresceins were analyzed with flow cytometry. The percentages of CD4 + CD8- (**a**) or CD4-CD8+ (**b**) T cells were calculated and sequentially displayed according to the order of culture time. The data of the uninfected group served as a control. The percentage of GFP+ cells in CD4+ or CD8+ T cell subsets was displayed to show the dynamic expression of GFP (**c**)

The viruses transduced T cell subgroups with different efficiency. They might also have different effects on the growth of T cell subsets. We observed the dynamics of CD4+ and CD8+ percentage in a period of 72 h after infection. As shown in Fig. 7a and b, the CD4+ percentage had a trend of decrease while the CD8+ cell proportion had a trend of increase as the culture time extended. Because cells in the uninfected group experienced exactly the same trends, it could be concluded that the change of CD4/CD8 ratio resulted from the inherent property of these cells or from the culture system but not from the virus infection.

Adenovirus is a vector for transient expression, we observed the dynamic expression of GFP in this experiment. As shown in Fig. 7c, the GFP+ percentage reached the peak 48 h post infection (hpi) and started to slightly decrease 72 hpi, which was in agreement with the feature of adenoviral transduction of rapidly growing cells.

### Effect of viral infection on cell growth

**Fig. 8:**
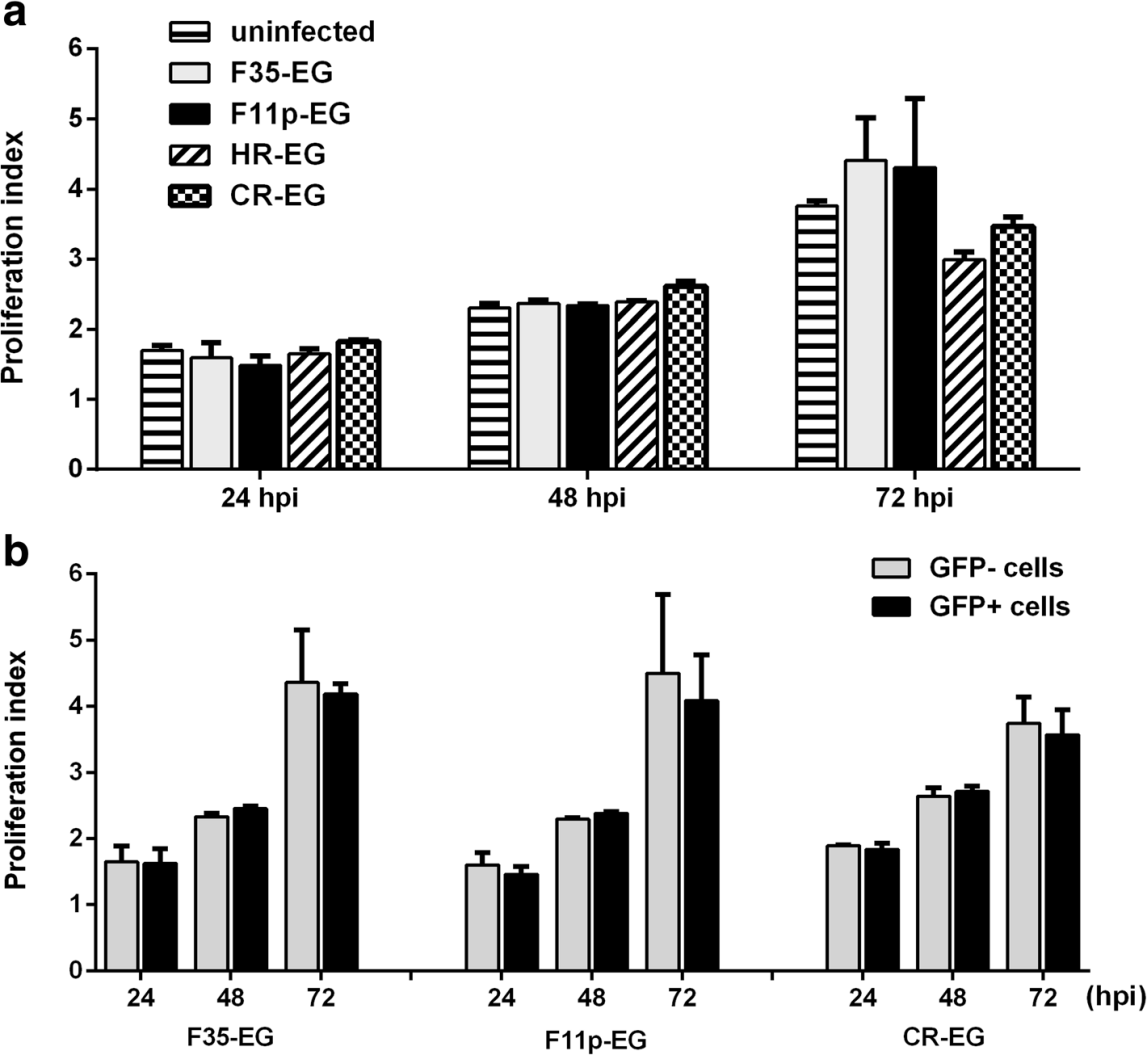
Effect of viral infection on the growth of T cells. T cells were isolated, expanded, stained with Dye eFluor 670, and infected by adenoviral vectors at an MOI of 1000 vp/cell. At 0, 24, 48 and 72 h post infection (hpi), cells were harvested and fixed in 1.5% paraformaldehyde in PBS. Fluorescence of GFP and eFluor 670 were analyzed with flow cytometry at the end of the experiment. Proliferation index (PI) of the total cells were calculated (**a**), or PI values of GFP- or GFP+ cells were separately calculated (**b**)

Infection of adenovirus at very high MOI would cause cytotoxicity to host cells. Effect of viral infection on T cell growth was investigated with the method of proliferation analysis. Proliferation index (PI) was the average number of cells that one original cell became. Compared with the uninfected T cells, cells which were infected with adenoviruses at an MOI of 1000 vp/cell showed similar PI values in the first 2 days (Fig. 8a). As the culture time extended to 3 days, the PI values displayed some volatility among uninfected and infected groups, which might result from experimental deviation. We chose to analyze the data in another way. There were GFP+ and GFP- cells in individual culture wells. Because GFP- cells could be treated as uninfected and serve as the internal control, we gated GFP- and GFP+ cells through flow cytometry analysis and separately calculated the PI value. As shown in Fig. 8b, the results indicated that the viral infection had little influence on T cell growth.

## Discussion

Hematopoietic cells are an important class of target cells for gene therapy. It is well-known that HAdV-B fiber pseudotyped HAdV-5 vectors could transduce hematopoietic cell lines, CD34+ blood cells and primary leukemia cells. However, the gene transfer ability of these vectors has not been thoroughly investigated in human T cells, the valuable target cells for immunocellular therapy in recent years. We compared HAdV-35 and HAdV-11p fiber pseudotyped HAdV-5 (F35-EG and F11p-EG), and found that both of them could transduce hematopoietic cells efficiently while F11p-EG showed slim advantage in most cell lines, CD34+ cord blood cells, and primary T cells. The interaction between RGD motif in HAdV penton and coreceptor integrin leads to the viral entry to host cells. Combination of fiber substitution and RGD-modification could have synergistic effect on viral gene transduction. Therefore, we added the RGD4C peptide to the HI loop or the C-terminus of HAdV-11p fiber knob to generate HR-EG or CR-EG viruses. RGD-modification of the HI loop of HAdV-11p fiber did increase the viral infection of HL-60 and 293 cells. However, HR-EG virus showed a significantly decreased ability to transduce U937, CD34+ and T cells. It is possible that incorporation of RGD4C peptide in HI loop impaired the interaction of the fiber with its original receptor although the modified fiber obtained the feature of binding new molecules. Fusing RGD4C to the C-terminus of fiber had negligible influence on gene transduction of hematopoietic cells. Because fiber C-terminus was a free end of the tertiary structure and located at the border, such modification retained integrity of the knob domain and applied no damage to the fiber-receptor interaction. However, C-terminal fusion of RGD4C brought no benefit for gene transfer, implying steric obstacles restricted RGD4C from binding integrins. Based on previous publications and our results, it could be concluded that the effect of RGD4C incorporation was dependent on the serotype of fiber knob, the length of fiber shaft and the position of insertion [26–29].

CMV promoter is widely applied to control transgene expression. However, it was reported that CMV promoter inclined to be silenced when used in hematopoietic cells. Transgene could be expressed at a very low level or could not be detected [28]. Activity of many other promoters has been tested in hematopoietic cells [30–34]. Human EF1a promoter contributes to an effective house-keeping expression. Replacing CMV promoter with that of EF1a dramatically enhanced the expression of target gene in cord blood CD34+ cells if we compared results here with that of our previous study [25]. HAdV5f11p-GFP with the CMV promoter could hardly transduced primary lymphoid leukemic cells while F11p-EG transduced 80% primary T cells at an MOI as low as 500 vp/cell (Fig. 5a), suggesting that vectors based on HAdV5F11p with the EF1a promoter could satisfy the reqirement of transient transduction of primary T cells.

Gene transduction of T cell by fiber-modified HAdV-5 was studied in detail in this study. We found that adenovirus could transduce more CD4+ cell than CD8+ cells (Fig. 6b), and viral infection did not change the ratio of CD4+/CD8+ (Fig. 7a and b). Furthermore, F11p-EG had no cytotoxicity when being used at an MOI as high as 1000 vp/cell where appoximately 80% T cells could be transduced (Fig. 8).

## Conclusions

HAdV-11p fiber pseudotyped HAdV-5 could transduce human primary T cells efficiently when the human EF1a promoter was used to control the expression of transgene. Such fiber-modified HAdV-5 has the potential to be used in T cell-based immunocellular therapy. Modification of the knob domain of HAdV-11p fiber with RGD motif did not further improve the gene transfer ability.

## Methods

### Donors

Six human peripheral blood samples and 3 human cord blood samples were obtained from anonymous adult donors after informed consent in accordance with the local ethics committee (Medical ethics committee of affiliated hospital of Qingdao university).

### Construction of adenoviral plasmids

The information of PCR primers and templates was summarized in Table 1. Adenoviral plasmids were constructed by modifying the AdEasy system (Fig. 1a and b). Firstly, the CMV promoter in pShuttle-CMV was replaced by human EF1a promoter to generate the shuttle plasmid pSh5EF1a. Fragments of Encapsidation signal (ES), EF1a promoter (EF1ap) and multiple cloning site (MCS) were amplified by PCR or obtained by self-annealing of two DNA oligos. The 3 fragments were combined and fused to form one DNA fragment (ES-EF1ap-MCS) by overlap extension PCR. ES-EF1ap-MCS was digested with BsrGI/EcoRV and inserted into the corresponding sites of pShuttle-CMV to generate pSh5EF1a. The coding sequence (CDS) of GFP gene was amplified by PCR and was inserted into the KpnI/HindIII site of pSh5EF1a to generate shuttle plasmid pSh5EF1a-GFP (shortened as pSh5EG). Secondly, the fiber gene in the backbone plasmid pAdEasy-1 was modified to generate new backbone plasmids of HAdV-5 vector. The plasmids pFiber5-11p and pAdEasy-F11p, which carrying chimeric fiber gene of HAdV-5 and HAdV-11p, were constructed previously [25]. As illustrated in Fig. 1b, DNA sequence encoding RGD4C peptide (CDCRGDCFC) was integrated into the XbaI-HIRGD-MfeI fragment by PCR and overlap extension PCR using primers and templates described in Table 1 [35, 36]. XbaI/MfeI digested PCR product was inserted into the corresponding sites of pFiber5-11p to generate pFiber5-11pHR. pFiber5-11pHR was digested with EcoRI, dephosphorized, and used to substitute the corresponding part of pAdEasy-1 to generate pAdEasy-F11pHR backbone plasmid. In pAdEasy-F11pHR, RGD4C was added to the HI loop of chimeric fiber of HAdV-5 and HAdV-11p. To add RGD4C peptide to the C-terminal of the chimeric fiber, similar operation was performed except that different PCR primers was designed and used (Table 1), and the generated backbone plasmid was named pAdEasy-F11pCR. In pAdEasy-F11pCR, [GGGGS]3 linker was used to connect RGD4C peptide with the C-terminus of the fiber [29, 37]. To construct backbone plasmid carrying chimeric fiber of HAdV-5 and HAdV-35, chimeric fiber gene was synthesized according to genbank AC_000019, digested with AgeI/MfeI and used to substitute the corresponding part of pFiber5-11p to generate pFiber5–35 plasmid [25]. The backbone plasmid pAdEasy-F35 was similarly constructed (Fig. 1b). Finally, adenoviral plasmids were generated with the method of homologous recombination by electroporating *E. coli* BJ5183 strain with backbone plasmid and linearized pSh5EG [38].

**Table 1:** summary of PCR information

Fragment	Primers code	Primers sequence	Template	Length of PCR product (bp)	restriction enzyme
ES	1411Sh5EF1aF1	ccggtgtaca caggaagtga caat	pShuttle	181	BsrGI
	1411Sh5EF1aR1	cttttgtatg aattactcga cgtcagtatt acgcgctatg agtaacacaa			AatII
EF1ap	1411Sh5EF1aF2	cgcgtaatac tgacgtcgag taattcatac aaaaggactc gc	pLVX-EF1a-Tet3G	1360	AatII
	1411Sh5EF1aR2	acggtacctc acgacacctg aaatggaaga a			KpnI
MCS	1411Sh5EF1aF3	ttccatttca ggtgtcgtga ggtaccgtcg acgcggccgc acgcgttcta	self-anneal	80	KpnI
	1411Sh5EF1aR3	ggccgatatc ttagctagca agcttaggtc tagaacgcgt gcggccgcgt			EcoRV
ES-EF1ap-MCS		overlap extension PCR		1558	
GFP	1703GFP-kf	ggccggtacc atggtgagca agggcgagga g	pLEGFP-C1	748	KpnI
	1703GFP-hr	ggccaagctt tagagtccgg acttgtacag ctcgt			HindIII
XbaI-HIRGD	1702F11pRGD1	ccagcacgac tgcctatcct tt	pFiber5-11p	164	XbaI
	1702F11pHIRGD2	gaaacagtct ccgcggcagt cacaatttat tgctcttcgg ttaagcatg			
HIRGD-MfeI	1702F11pHIRGD3	tgtgactgcc gcggagactg tttctgcgac gagacatcat attgtattcg tataac	pFiber5-11p	240	
	1702F11pRGD4	ctgaatgaaa aatgacttga aattttct			MfeI
XbaI-HIRGD-MfeI		overlap extension PCR		380	
XbaI-CRGD	1702F11pRGD1	ccagcacgac tgcctatcct tt	pFiber5-11p	284	XbaI
	1702F11pCRGD2	tgaaccgcca ccacctgagt cgtcttctct gatgtagtaa aaggta			
CRGD	1702F11pCRGD3	gaagacgact caggtggtgg cggttcaggc ggaggtggct ctggcggtgg cggat	self-anneal	90	
	1702F11pCRGD4	ggctcagcag aaacagtctc cgcggcagtc acacgatccg ccaccgccag agcca			
CRGD-MfeI	1702F11pCRGD5	cgcggagact gtttctgctg agcccaagaa taaagaatcg	pFiber5-11p	105	
	1702F11pRGD4	ctgaatgaaa aatgacttga aattttct			MfeI
XbaI-CRGD-MfeI		overlap extension PCR		428	

### Cell culture

The cell line 293 (ATCC no. CRL-1573) was cultured in Dulbecco’s modified Eagle’s medium (DMEM) plus 8% fetal bovine serum (FBS; HyClone, Logan, UT, USA). Human leukemic cell lines U937 (promonocytic leukemia), K562 (chronic myelogenous leukemia), Jurkat (T-cell leukemia), and HL-60 (acute myelogenous leukemia) were cultured with RPMI 1640 medium plus 10% FBS. All cells were maintained at 37 °C with 5% CO_2_ in a humidified incubator and regularly split every 3 to 4 days.

### Cord blood CD34+ cell isolation

Mononuclear cells (MNCs) were harvested from fresh buffy coats by Ficoll-Paque density gradient separation from pooled human cord blood samples of healthy donors. Medical ethics committee of affiliated hospital of Qingdao university approved all of the experiments. CD34+ cells were isolated from MNCs by using a CD34+ progenitor cell positive isolation kit (CD34 MicroBead Kit, CAT# 130–046-703; Miltenyi Biotech). Purity was routinely > 95% as assessed by flow cytometric analysis. CD34+ cells were maintained in serum-free medium (StemSpan SFEM, CAT#09650; Stemcell Technologies) supplemented with cytokine cocktail (50 ng/ml interleukin-3; 100 ng/ml interleukin-6; 100 ng/ml Flt-3 ligand; 50 ng/ml stem cell factor and 100 ng/ml thrombopoietin). Two days after isolation, cells were infected with adenoviral vectors.

### Human T cell isolation

MNCs were collected from fresh buffy coats by Ficoll-Paque density gradient separation from peripheral blood samples of healthy donors. Medical ethics committee of affiliated hospital of Qingdao university approved all of the experiments.T cells were isolated from MNCs by using a T cell negative isolation kit (Dynabeads Untouched Human T Cells Kit, CAT#11344D; Life Technologies). Isolated T cells were cultured in X-VIVO 15 medium (CAT#04-418Q; Lonza) supplemented with 10% FBS (CAT#ASM-5007; Applied StemCell) and 400 IU/ml rIL-2 (Beijing SL Pharmaceutical) and expanded by incubating with Dynabeads Human T-Activator CD3/CD28 according to the manufacturer’s instructions (CAT#11131D; Life Technologies). Expanded T cells were maintained in X-VIVO 15 medium plus 10% FBS and 2000 IU/ml rIL-2, and used for viral infection 8 to 14 days after isolation.

### Preparation of adenoviral vectors

Adenoviral plasmids were digested with PacI, recovered by ethanol precipitation and used to transfect 293 cells with Lipofectamine 3000 according to the manufacturer’s instructions (Life technologies). Plaques occurred within 1 week post transfection. Rescued viruses was released by three rounds of freeze-and-thaw and amplified in 293 cells. Amplified virus was purified with the traditional method of CsCl ultracentrifugation. Particle titer was determined by quantifying the genomic DNA of purified virus, and the infectious titer was determined by limiting dilution assay on 293 cells [39].

### Transduction of hematopoietic cells

Exponentially proliferating cells were counted with hemacytometer, diluted and seeded in 24-well (for cell lines) or 96-well plates (for CD34+ or T cells) with a density of 3 × 10^5^ cell/well. Purified viruses were diluted with culture medium and added to each well in a volume to achieve indicated multiple MOI. The infection volume was adjusted to the half amount of routine culture, which was 0.25 ml for each well in 24-well plate and 0.1 ml for 96-well plate, respectively. The plates were transferred to cell culture incubator, and fresh medium in the half volume of routine culture was supplemented to each well without removal of virus 24 hpi unless otherwise indicated.

### Flow cytometry analysis

Following antibodies were used in flow cytometry assay: APC Mouse Anti-Human CD3 (CAT#555335; BD Pharmingen), PerCP-Cyanine5.5 CD4 Monoclonal Antibody (OKT4, CAT#45–0048-42; Thermo Fisher Scientific), PE CD8a Monoclonal Antibody (OKT8, CAT#12–0086-42; Thermo Fisher Scientific), and APC CD34 Monoclonal Antibody (4H11,CAT# 17–0349-42; Thermo Fisher Scientific). Cells were transferred to 1.5-ml Eppendorf tube, washed once with PBS containing 1% FBS, labelled with fluorescein-conjugated antibodies for 20 min at room template, washed with and then suspended in PBS containing 1% FBS, and analyzed by flow cytometry.

### Cell proliferation analysis

Dye eFluor 670 (CAT#65–0840; Thermo Fisher Scientific) was used to label T cells for proliferation analysis according to the manufacturer’s instructions. Labelled cells were mixed with viruses and aliquotted to wells in 96-well plate at a density of 2 × 10^5^ cell/well. Fresh medium of 100 μl was added to each well 24 h later. At indicated time points, cells were washed with and suspended in PBS containing 1% FBS, dispersed into singles cells, supplemented with 4% paraformaldehyde in PBS to a final concentration of 1.5%, and reserved at 4 °C. After going through all the time points, fixed cells were analyzed with flow cytometry. PI, which was defined as the number of modeled cells (the number of cells at the time point of harvest) divided by the cells in the original culture (the number of cells at the time of seeding), was calculated with the software of ModFit LT (Verity Software House).

### Statistical analysis

The Data were represented as the mean ± standard deviation (SD) of representative experiments. The statistical analysis was performed using the Analysis of Variance (ANOVA) test. A *p*-value less than 0.05 was considered to be significant.

## Abbreviations

ANOVA: Analysis of Variance
CDS: Coding sequence
DMEM: Dulbecco’s modified Eagle’s medium
EBNA-1: EBV nuclear antigen 1
EF1ap: EF1a promoter
ES: Encapsidation signal
HAdV: Human adenoviruses
HAdV-5: Human adenovirus 5
Hpi: Hours post infection
MCS: Multiple cloning site
MNCs: Mononuclear cells
MOI: Multiplicity of infection
PI: Proliferation index
S/MAR: Scaffold/matrix attachment region
SD: Standard deviation
vp/cell: Viral particle per cell
